# Investigating the effects of microstructural changes induced by myocardial infarction on the elastic parameters of the heart

**DOI:** 10.1007/s10237-023-01698-2

**Published:** 2023-03-03

**Authors:** Laura Miller, Raimondo Penta

**Affiliations:** School of Mathematics and Statistics, University of Glasgow, University Place, Glasgow, G12 8QQ UK

**Keywords:** Poroelasticity, Computational modelling, Asymptotic homogenization, Myocardial infarction

## Abstract

Within this work, we investigate how physiologically observed microstructural changes induced by myocardial infarction impact the elastic parameters of the heart. We use the LMRP model for poroelastic composites (Miller and Penta in Contin Mech Thermodyn 32:1533–1557, 2020) to describe the microstructure of the myocardium and investigate microstructural changes such as loss of myocyte volume and increased matrix fibrosis as well as increased myocyte volume fraction in the areas surrounding the infarct. We also consider a 3D framework to model the myocardium microstructure with the addition of the intercalated disks, which provide the connections between adjacent myocytes. The results of our simulations agree with the physiological observations that can be made post-infarction. That is, the infarcted heart is much stiffer than the healthy heart but with reperfusion of the tissue it begins to soften. We also observe that with the increase in myocyte volume of the non-damaged myocytes the myocardium also begins to soften. With a measurable stiffness parameter the results of our model simulations could predict the range of porosity (reperfusion) that could help return the heart to the healthy stiffness. It would also be possible to predict the volume of the myocytes in the area surrounding the infarct from the overall stiffness measurements.

## Introduction

Poroelasticity has been applied to many biological scenarios that comprise an elastic matrix with an interconnected fluid flow to investigate their effective mechanical behaviour. The theory was first developed by Biot (1955, 1956a, b, 1962a) and it is applicable to situations where the interactions between the solid and the fluid take place at a scale much smaller than the overall tissue scale. The theory can be applied to a variety of materials including hard hierarchical tissues such as the bones and the tendons (Cowin 1999; Weiner & Wagner 1998) and also to soft biological tissues such as the interstitial matrix, tumours (Bottaro and Ansaldi 2012; Flessner 2001) and also the myocardium of the heart (May-Newman and McCulloch 1998; Cookson et al. (2012; Chapelle et al. 2010; Bukac et al. (2015).

The effective mechanical behaviour of a material can be derived via a variety of homogenization techniques. Their is to incorporate the porescale interactions and properties into the effective macroscale behaviour of materials. Without using this type of modelling, it would be computationally impossible to resolve all of the porescale details. These techniques, see, e.g., Mei and Vernescu (2010), Auriault et al. (2010), and Holmes (2012) include volume averaging, mixture theory, and asymptotic homogenization. A comparison of these techniques can be found in Hori and Nemat-Nasser (1999) and Davit et al. (2013).

The asymptotic homogenization technique exploits the scale separation present in material systems to fully decouple spatial scales and derive a macroscale model where the coefficients encode the microstructural details. This technique has been applied to poroelastic materials by Burridge and Keller (1981), Wang (2017), Lévy (1979) and Penta et al. (2020). The theory has since been extended to model a vast range of scenarios including growth of poroelastic materials (Penta et al. 2014), vascularised poroelastic materials (Penta and Merodio 2017) and poroelastic composites (Miller and Penta 2020). Recently there has also been a development of the theory for nonlinear poroelastic materials (Collis et al. 2017; Brown et al. 2014; Ramírez-Torres et al. 2018) and nonlinear poroelastic composites (Miller and Penta 2021a). The theory has also been investigated with various additional scales such as poroelastic with inclusion (Royer et al. 2019) and Chen et al. (2018) and double poroelastic (Miller and Penta 2021b).

In Miller and Penta (2020) the authors develop a novel multiscale model for poroelastic composites. This was then extended in Miller and Penta (2022) where a robust 2D and 3D computational platform was developed. Within this work we will use this platform to perform simulations to determine the elastic parameters of the heart.

The human heart has four chambers each of which have a muscular wall with three distinct layers, the endocardium, the myocardium, and the epicardium. The endocardium and epicardium are the thin inner and outer layers, whereas the myocardium is the middle and most dominant layer. It is supplied by the coronary arteries and is the layer most affected by a variety of diseases, e.g., myocardial infarction, angina and the effects of ageing (Whitaker 2014; Weinhaus and Roberts 2005).

The myocardium has a structure where there are cardiac myocytes (muscle cells) embedded in a collagen matrix, which is produced by the cardiac fibroblasts, with an interconnected fluid (blood) flow through permeating vasculature (Purslow 2008). These structures are visible on a microscale length which is much smaller than the size of the heart muscle. The myocardium microstructure is complex geometrically and is strongly impacted by a variety of diseases, in particular myocardial infarction (heart attack). In the case of myocardial infarction blood flow is reduced to an area of myocardium tissue, this results in the death of the cardiac myocytes and in their place, we find collagen rich scar tissue produced by the fibroblasts to retain the structural integrity of the myocardium (Fan et al. 2012; Humeres and Frangogiannis 2019). The size and amount of scar tissue affect the heart’s functionality post recovery (Ertl and Frantz 2005). As a result of the loss of cardiac myocytes, the remaining myocytes in the area surrounding the infarct increase in volume to attempt to retain homeostasis in the heart (Kozlovskis et al. 1991). The increase in the volume of the myocytes corresponds to the infarct size (Olivetti et al. 1987, 1994; Anversa et al. 1985).

There have been a variety of approaches taken to model the heart summarised in the review articles (Peirlinck et al. 2021; Owen et al. 2018; Smith et al. 2004). The most prominent of these include constitutive non-linear elastic approaches using the Holzapfel-Ogden Law (Holzapfel and Ogden 2009). The work (Holzapfel and Ogden 2009) describes the myocardium as a non-homogeneous, nonlinear elastic and incompressible material and then proposes a general theoretical framework that uses invariants associated with the three orthogonal directions that can be identified within the material. This work has paved the way for a variety of extensions in an attempt to understand the phenomena of the heart behaviour such as in Guan et al. (2019) and Wang et al. (2014), and different methods of numerical implementation such as Pezzuto et al. (2014). A viscoelastic approach to understanding the myocardium has also been taken by Gültekin et al. (2016) and Nordsletten et al. (2021). Within these works there is the aim to address the viscoelastic phenonmena observed experimentally by modifying the constitutive laws previously used for the myocardium. There has also been a poroelastic approach taken by May-Newman and McCulloch (1998); Cookson et al. (2012) and Chapelle et al. (2010). This approach aims to incorporate the porescale fluid flow into the overall behaviour of the myocardium and to consider the perfused muscle.

Within this work, we aim to investigate the effects of microstructural changes induced by myocardial infarction on the elastic parameters of the heart. In Sect. 2 we summarise the LMRP (Miller and Penta 2020) model for poroelastic composites which we will use to model the microstructure of the myocardium. Within the sections that follow we will investigate a variety of changes to the parameters and geometry of the microstructure in order to simulate a variety of phenomena observed post myocardial infarction. We account for the anisotropy of the heart microstructure through the inclusion of the myocytes in one direction. In Sect. 3, we will investigate the comparison between healthy elastic parameters and the parameters obtained in the post myocardial infarction setting of loss of myocyte and increased fibrosis. Then in Sect. 4, we consider the effect that the increase in myocyte volume fraction has on the elastic parameters of the myocardium post myocardial infarction. Finally in Sect. 5, we propose a 3D framework to model the myocytes connected via intercalated disks. We conclude this work by providing the future prospects of developing this model and its potential as a diagnostic tool to aid clinicians.

## The mathematical model

**Fig. 1:**
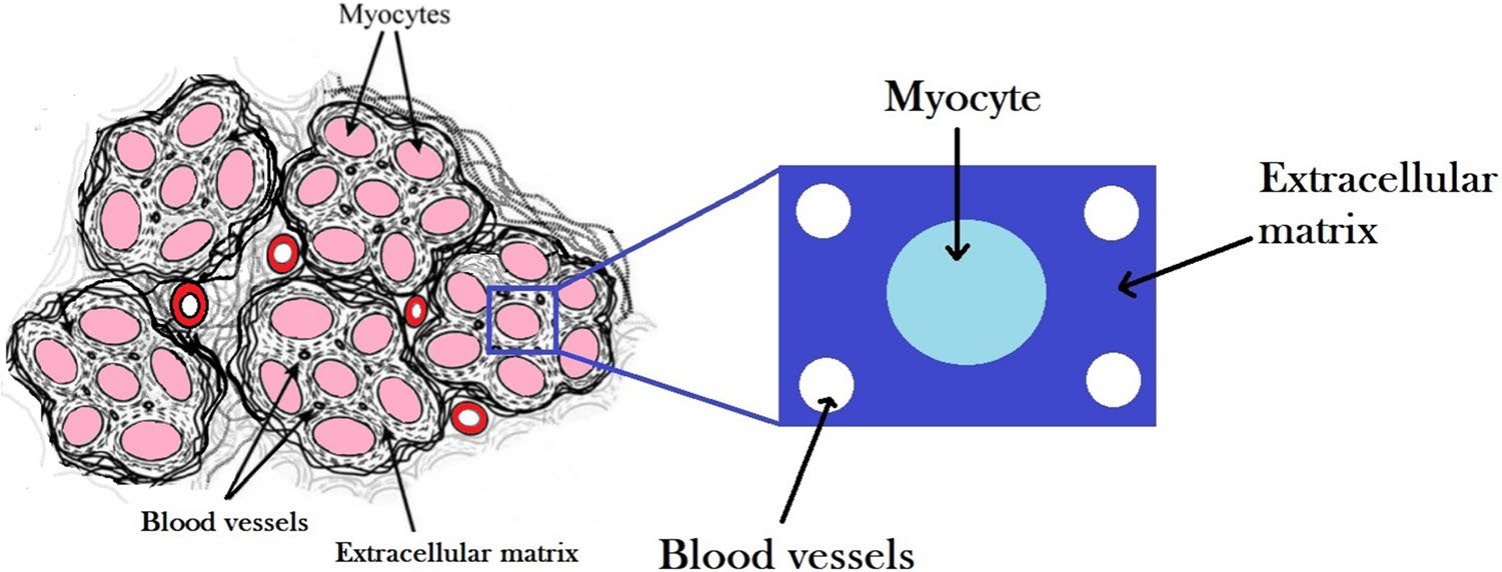
Image of heart microstructure and the assumed microstructural geometry of our model

We use the LMRP model for poroelastic composites (Miller and Penta 2020) to describe the microstructure of the myocardium tissue. The myocardium is predominantly comprised of an extracellular matrix with embedded blood vessels and cardiac myocyte cells. We, therefore, have two elastic phases and a fluid interacting on the microscale, see (Fig. 1).

Here, we summarise the mathematical model for a poroelastic composite derived by the asymptotic homogenization technique in Miller and Penta (2020) that we will use to describe the myocardium microstructure. The model derivation is carried out by setting up an appropriate fluid-structure interaction problem between a linear elastic porous matrix, $\Omega _{\scriptscriptstyle \textrm{II}}$, with embedded linear elastic subphases, $\Omega _{\scriptscriptstyle \textrm{I}}$, with a Newtonian fluid, $\Omega _{\scriptscriptstyle \textrm{f}}$, flowing in the pores. When applying this to the myocardium we make the identifications that $\Omega _{\scriptscriptstyle \textrm{II}}$ is the extracellular matrix, $\Omega _{\scriptscriptstyle \textrm{I}}$ is the myocyte and $\Omega _{\scriptscriptstyle \textrm{f}}$ is the permeating vasculature of the heart. Before describing the model equations we wish to clarify the notation that we will use throughout the work.

### Remark 1

(Notation) We use the following for a generic field. For a scalar, we use ordinary lowercase letters e.g. *v*, for a vector we use boldface e.g. $\textbf{v}$, then ${\textsf{V}}$ is used for second rank tensor. We also use uppercase normal text e.g. *V* for third rank tensors, and finally ${\mathbb {V}}$ is used for fourth rank tensors. There are some exceptions to this notation to keep the style consistent with classical notation used for the Biot’s modulus and the Biot’s tensor of coefficients as well as porosity. In these cases the Biot’s modulus is *M*, the Biot’s tensor is $\varvec{\alpha }$ and the porosity is $\phi$ as used in Biot (1962b); Burridge and Keller (1981).

The fluid-structure interaction problem comprises a balance equation for each of the solid domains and the fluid domain. We can write these as

1 $$\begin{aligned} \nabla \cdot {\textsf{T}}_{\scriptscriptstyle \textrm{Myo}}=0&\quad \text{ in } \quad \Omega _{\scriptscriptstyle \textrm{I}},\end{aligned}$$

2 $$\begin{aligned} \nabla \cdot {\textsf{T}}_{\scriptscriptstyle \textrm{M}}=0&\quad \text{ in } \quad \Omega _{\scriptscriptstyle \textrm{II}},\end{aligned}$$

3 $$\begin{aligned} \nabla \cdot {\textsf{T}}_{\scriptscriptstyle \textrm{f}}=0&\quad \text{ in } \quad \Omega _{\scriptscriptstyle \textrm{f}}, \end{aligned}$$

where ${\textsf{T}}_{\scriptscriptstyle \textrm{Myo}}$, ${\textsf{T}}_{\scriptscriptstyle \textrm{M}}$ and ${\textsf{T}}_{\scriptscriptstyle \textrm{f}}$ are the stress tensors in the myocyte, extracellular matrix and the fluid respectively. The solid phases are linear elastic and the fluid is incompressible and Newtonian so the constitutive laws for each of these phases are given as

4 $$\begin{aligned} {\textsf{T}}_{\scriptscriptstyle \textrm{Myo}}={\mathbb {C}}_{\scriptscriptstyle \textrm{Myo}}\nabla \textbf{u}_{\scriptscriptstyle \textrm{Myo}}&\quad \text{ in } \quad \Omega _{\scriptscriptstyle \textrm{I}},\end{aligned}$$

5 $$\begin{aligned} {\textsf{T}}_{\scriptscriptstyle \textrm{M}}={\mathbb {C}}_{\scriptscriptstyle \textrm{M}}\nabla \textbf{u}_{\scriptscriptstyle \textrm{M}}&\quad \text{ in } \quad \Omega _{\scriptscriptstyle \textrm{II}},\end{aligned}$$

6 $$\begin{aligned} {\textsf{T}}_{\scriptscriptstyle \textrm{f}}=-p{\textsf{I}}+\mu \nabla \textbf{v}+(\nabla \textbf{v})^{\textrm{T}}&\quad \text{ in } \quad \Omega _{\scriptscriptstyle \textrm{f}}, \end{aligned}$$

where ${\mathbb {C}}_{\scriptscriptstyle \textrm{Myo}}$ and ${\mathbb {C}}_{\scriptscriptstyle \textrm{M}}$ are the fourth-rank elasticity tensor in the myocyte and the extracellular matrix; $\textbf{u}_{\scriptscriptstyle \textrm{Myo}}$ and $\textbf{u}_{\scriptscriptstyle \textrm{M}}$ are the elastic displacements in each of the phases, *p* is the fluid pressure, $\textbf{v}$ is the fluid velocity and $\mu$ is the fluid viscosity. Since the fluid is incompresible we have the incompressibility constraint given as

7 $$\begin{aligned} \nabla \cdot \textbf{v}=0 \quad \text{ in } \quad \Omega _{\scriptscriptstyle \textrm{f}}. \end{aligned}$$

The fluid structure interaction problem is then to be closed by appropriate interface conditions. These are continuity of tractions and elastic displacements between the myocytes and the extracellular matrix

8 $$\begin{aligned} {\textsf{T}}_{\scriptscriptstyle \textrm{Myo}}\textbf{n}_{\scriptscriptstyle\mathrm{III}}={\textsf{T}}_{\scriptscriptstyle \textrm{M}}\textbf{n}_{\scriptscriptstyle\mathrm{III}}&\quad \textrm{on} \quad \Gamma_{\scriptscriptstyle\mathrm{III}},\end{aligned}$$

9 $$\begin{aligned} {\textbf{u}}_{\scriptscriptstyle \textrm{Myo}}={\textbf{u}}_{\scriptscriptstyle\mathrm{M}}&\quad \textrm{on} \quad \Gamma_{\scriptscriptstyle\mathrm{III}}, \end{aligned}$$

and the continuity of tractions and velocities between the extracellular matix and the fluid flowing in the vessels,

10 $$\begin{aligned} {\textsf{T}}_{\scriptscriptstyle \textrm{M}}\textbf{n}_{\scriptscriptstyle\mathrm{II}}&={\textsf{T}}_{\scriptscriptstyle \textrm{f}}\textbf{n}_{\scriptscriptstyle\mathrm{II}}\quad \textrm{on} \quad \Gamma_{\scriptscriptstyle\mathrm{II}},\end{aligned}$$

11 $$\begin{aligned} \dot{\textbf{u}}_{\scriptscriptstyle \textrm{M}}&={\textbf{v}}\quad \textrm{on} \quad \Gamma_{\scriptscriptstyle\mathrm{II}}, \end{aligned}$$

where $\mathbf{n}_{\scriptscriptstyle\mathrm{II}}$ and $\mathbf{n}_{\scriptscriptstyle\mathrm{III}}$ are the unit outward normal vectors to the fluid-solid and solid-solid interfaces $\Gamma_{\scriptscriptstyle\mathrm{II}}$ and $\Gamma_{\scriptscriptstyle\mathrm{III}}$, respectively. The Eqs. (1)–(11) form our complete fluid structure interaction problem.

We make the assumption that the radius of the blood vessels, *d* (the porescale), is comparable with the distance between the adjacent myocytes (Potter and Groom 1983; Tracy 2014). Overall this length is much smaller than the size of the entire myocardium, *L* (the macroscale). The difference in length scales is the scale separation parameter $\epsilon$. This allows us to introduce two variables, one is $\textbf{x}$ for the macroscale, and one to capture the microscale variations $\textbf{y}$. Having this difference in lengths allows us to decouple the spatial scales and apply the asymptotic homogenization technique to derive the macroscale model. We make the assumptions of microscale periodicity (Burridge and Keller 1981) and macroscopic uniformity (Penta et al. 2014; Holmes 2012). These assumptions mean that at each macroscale point we see the same repeating microstructure.

The asymptotic homogenization technique involves applying a multiple scales expansion and expressing each of the fields appearing in the fluid structure interaction problem as a power series in the scale separation parameter $\epsilon$. We then are able to equate coefficients of $\epsilon$ in the multiple scales expansion to derive the governing equations and to form differential problems with linear ansatz that lead to the microscale periodic cell problems that are to be solved to find the macroscale model coefficients. The cell problems that are used to find the model coefficients are detailed in the Appendix A.

The new system of partial differential equations is of poroelastic-type. The model equations contain coefficients that encode the properties of the underlying material microstructure such as the stiffness and geometry of the myocytes and extracellular matrix and the geometry of the channels.

Here, we summarise the four governing equations. The first macroscale equation is the balance of linear momentum

12 $$\begin{aligned} \nabla _{{\textbf{x}}} \cdot {\textsf{T}}_{\textrm{Eff}}=0, \end{aligned}$$

where we have the constitutive law

13 $$\begin{aligned} {\textsf{T}}_{\textrm{Eff}}=\langle \mathbb {C_{\scriptscriptstyle \textrm{Myo}}M_{\scriptscriptstyle \textrm{Myo}}} +\mathbb {C_{\scriptscriptstyle \textrm{Myo}}}+ \mathbb {C_{\scriptscriptstyle \textrm{M}}M_{\scriptscriptstyle \textrm{M}}} +\mathbb {C_{\scriptscriptstyle \textrm{M}}}\rangle _s\xi _x\textbf{u}^{(0)}+\varvec{\gamma }p^{(0)}, \end{aligned}$$

where ${\mathbb {C}}_i$ with $i=\textrm{Myo, M}$ is the elasticity tensor for the myocyte and matrix respectively. We can define the effective elasticity tensor $\widetilde{{\mathbb {C}}}$ as

14 $$\begin{aligned} \widetilde{{\mathbb {C}}}=\langle \mathbb {C_{\scriptscriptstyle \textrm{Myo}}M_{\scriptscriptstyle \textrm{Myo}}} +\mathbb {C_{\scriptscriptstyle \textrm{Myo}}}+ \mathbb {C_{\scriptscriptstyle \textrm{M}}M_{\scriptscriptstyle \textrm{M}}} +\mathbb {C_{\scriptscriptstyle \textrm{M}}}\rangle _s, \end{aligned}$$

The stress balance equation and constitutive law arrise from the summation of the asymptotic expansion of the stress balance equations.

The system also comprises the conservation of mass equation which we derive via the asymptotic expansion of the incompressibility constraint and the use of the ansatz to the elastic differntial problem. We have

15 $$\begin{aligned} \frac{{\dot{p}}^{(0)}}{M}=-\nabla _{{\textbf{x}}} \cdot \langle \textbf{w}\rangle _{f}-{\varvec{\alpha }}:\xi _{{\textbf{x}}}\dot{\textbf{u}}^{(0)}, \end{aligned}$$

where we have that $p^{(0)}$ is the macroscale pressure, $\dot{\textbf{u}}^{(0)}$ is the leading order solid velocity, $\textbf{w}$ is the average fluid velocity, *M* and ${\varvec{\alpha }}$ are the resulting Biot’s modulus and tensor of coefficients associated with the system respectively. The final macroscale equation is Darcy’s law

16 $$\begin{aligned} \langle \textbf{w}\rangle _f=-\langle {\textsf{W}}\rangle _f\nabla _{{\textbf{x}}}p^{(0)}, \end{aligned}$$

where $\langle {\textsf{W}} \rangle _f$ is the hydraulic conductivity tensor.

From our governing equations, we have that the behaviour of the poroelastic composite material (myocardium) can be fully characterised by the model coefficients, that is, by the effective elasticity tensor $\widetilde{{\mathbb {C}}}$, the hydraulic conductivity $\langle {\textsf{W}}\rangle _f$, the Biot’s tensor of coefficients ${\varvec{\alpha }}$ and the Biot’s coefficient *M*. These coefficients can be written as

17 $$\begin{aligned} {\varvec{\alpha }}=\phi \textbf{I}-\langle \text{ Tr }(&{\mathbb {M}}_{\scriptscriptstyle \textrm{Myo}}+{\mathbb {M}}_{\scriptscriptstyle \textrm{M}})\rangle _s, \quad M=\frac{-1}{\langle \text{ Tr }({\textsf{Q}}_{\scriptscriptstyle \textrm{Myo}}+{\textsf{Q}}_{\scriptscriptstyle \textrm{M}})\rangle _s}, \nonumber \\&\varvec{\gamma }=\langle \mathbb {C_{\scriptscriptstyle \textrm{Myo}}}{\textsf{Q}}_{\scriptscriptstyle \textrm{Myo}}+\mathbb {C_{\scriptscriptstyle \textrm{M}}}{\textsf{Q}}_{\scriptscriptstyle \textrm{M}}\rangle _s-\phi \textbf{I}, \end{aligned}$$

where the fourth rank tensors ${\mathbb {M}}_{\scriptscriptstyle \textrm{Myo}}$, ${\mathbb {M}}_{\scriptscriptstyle \textrm{M}}$ and the second rank tensors ${\textsf{Q}}_{\scriptscriptstyle \textrm{Myo}}$, ${\textsf{Q}}_{\scriptscriptstyle \textrm{M}}$ are to be computed by solving the microscale cell problems arising from the asymptotic homogenization. The asymptotic homogenization technique provides six elastic type cell problems that are to be solved to compute the strains ${\mathbb {M}}_{\scriptscriptstyle \textrm{Myo}}$, ${\mathbb {M}}_{\scriptscriptstyle \textrm{M}}$. These can then be used, along with the original input elasticity tensors for the material ${\mathbb {C}}_{\scriptscriptstyle \textrm{Myo}}$, ${\mathbb {C}}_{\scriptscriptstyle \textrm{M}}$ to compute the effective elasticity tensor. To see these elastic type problems explicitly see Appendix A and for even further details consider the references therein. The asymptotic homogenization technique also gives rise to a further vector problem that can be solved to obtain the tensors ${\textsf{Q}}_{\scriptscriptstyle \textrm{Myo}}$ and ${\textsf{Q}}_{\scriptscriptstyle \textrm{M}}$. By solving the seven problems we obtain the four tensors required so that we can compute all the coefficients of our novel macroscale model.

Within this work, since our analysis will focus predominantly on the elastic parameters of the myocardium in both healthy and diseased scenarios we will only compute the necessary components of the effective elasticity tensor $\widetilde{{\mathbb {C}}}$.

Lastly, we note the notation $\langle \psi \rangle$, which is a cell average defined as

18 $$\begin{aligned} \langle \psi \rangle _k=\frac{1}{|\Omega |}\int _{\Omega _k} \psi (\mathbf{{x,y}},t) \textrm{d}{} \mathbf{{y}} \quad k={f, s} \end{aligned}$$

where $\langle \psi \rangle _{\textrm{s}}=\langle \psi \rangle _{\textrm{M}}+\langle \psi \rangle _{\textrm{Myo}}$, and where $\psi$ is a general field in our system and $|\Omega |$ is the volume of the domain and the integration is taken over the porescale.

Before considering the numerical simulations for this model, we wish to make a few remarks on the well-posedness of the model and the cell problems. The novel model for poroelastic composites (Miller and Penta 2020), on which the simulations in this work are based, also contains rigorous results concerning well-posedness. In particular, the effective elasticity tensor (14) is proved to be positive-semi definite. This property can be proved using the cell problems properties and the definition of the tensor (14), Miller and Penta (2020). A similar proof is carried out in Penta and Gerisch (2017) who proved that the effective elasticity tensor for elastic composites was positive definite.

By proving the positive semi-definiteness of the effective elasticity tensor (14), along with other results proved in Miller and Penta (2020) (positive Biot Modulus and the equality between the Biot’s tensor of coefficients $\varvec{\alpha }$ and the coefficient $\varvec{\gamma }$ in Eq. (17)), we show that the model is of Biot-type, and therefore well-posed.

## Loss of myocytes and increased fibrosis

**Fig. 2:**
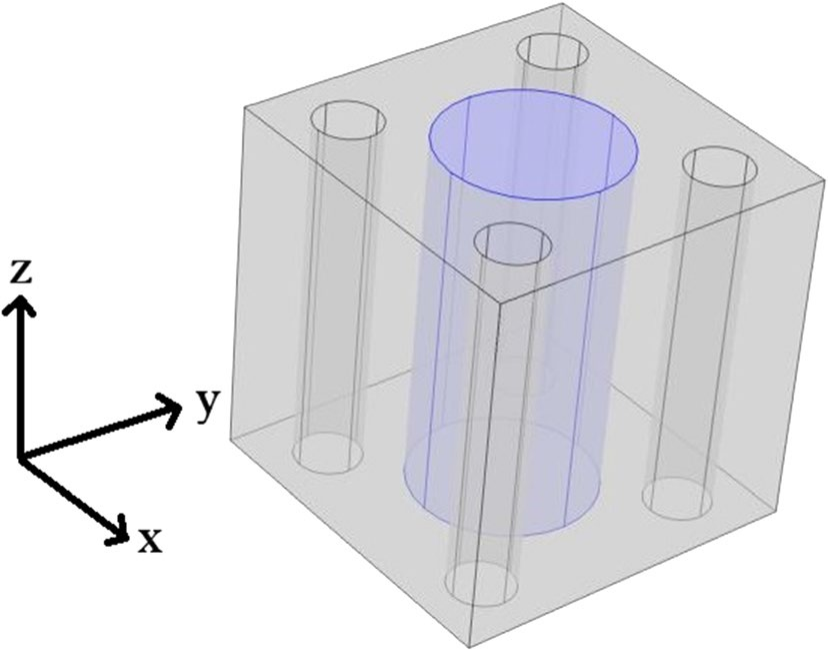
3D geometry healthy intact myocyte embedded in soft extracellular matrix with four blood vessels

**Fig. 3:**
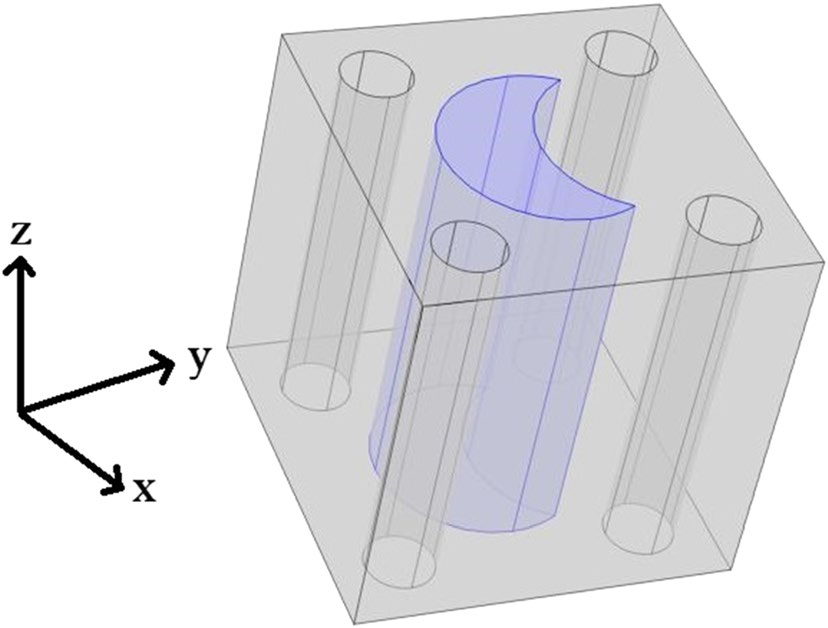
3D geometry myocyte that has been injured as a result of infarction embedded in the stiffer collagen rich extracellular matrix with four blood vessels

Within this section, we wish to compare the elastic parameters (Young’s and shear moduli) for the healthy myocardium versus the infarcted myocardium. The healthy myocardium is proposed to consist of a number of cardiac myocytes embedded in an extracellular matrix surrounded by a network of blood vessels supplying the myocytes (Purslow 2008). This structure is shown in Fig. 2. In the infarct region, we have a loss of myocytes due to the interruption in the blood flow supplying them which causes them to die or become damaged. In order to retain the structural integrity of the heart the extracellular matrix forms a collagen rich scar to replace the damaged and lost myocytes (Prabhu and Frangogiannis 2016). In order to simulate this myocyte damage, we have created the geometry, Fig. 3 where the myocyte is missing a section and we increase the stiffness of the extracellular matrix. The parameters chosen are shown in (Table 1).

**Fig. 4:**
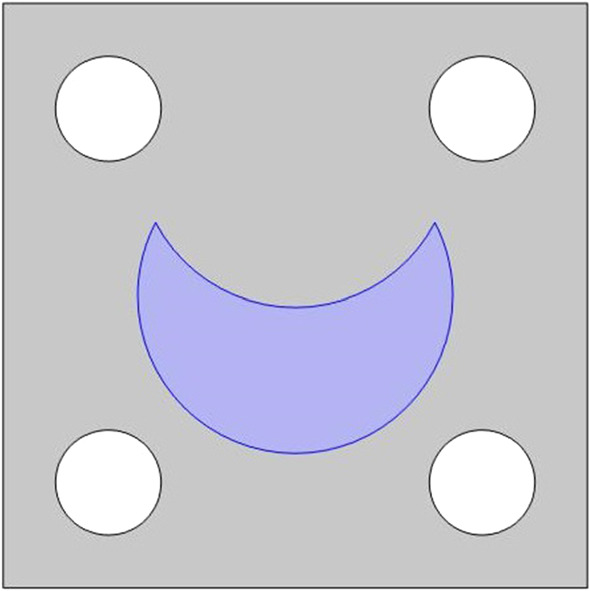
2D cross-section showing a myocyte that has been injured as a result of infarction embedded in the stiffer collagen rich extracellular matrix with four blood vessels

**Fig. 5:**
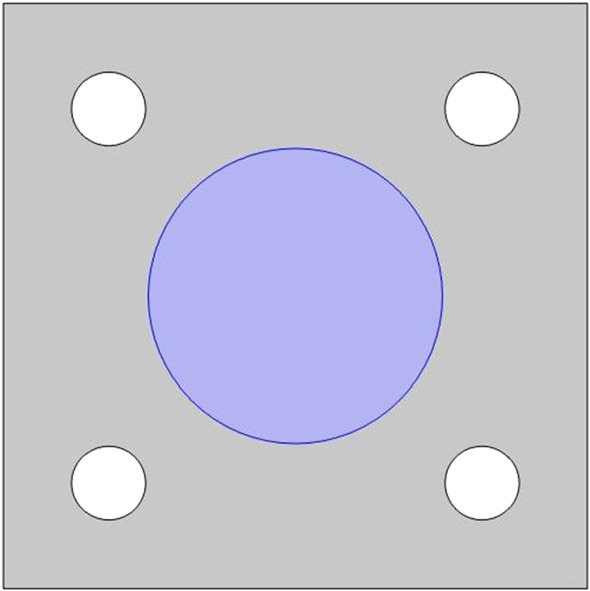
2D geometry for healthy myocyte embedded in the healthy extracellular matrix with four blood vessels

Within this section, we make the assumption that both the healthy and the damaged myocytes run from the top of the cell to the bottom as a single fibre. This means that we can cut the plane and perform 2D simulations (See Figs. 4, 5 for 2D geometry) to solve the cell problems of LMRP. The details of the 3D cell problems can be found in the Appendix A and the reduction of these problems to 2D can be found in Miller and Penta (2022).

We use the input parameters found in Table 1 to carry out our simulations. These parameters come from a variety of sources (Andreu et al. 2014; Chiou et al. 2016; Lieber et al. 2004).

**Table 1:** Input parameters

Model	$E_{\textrm{myo}}$	$E_{\textrm{matrix}}$	$\nu _{\textrm{myo}}$	$\nu _{\textrm{matrix}}$
Healthy	35	40	0.49	0.4
Infarcted	35	80	0.49	0.4

Due to the geometry, we are assuming for the microstructure we are including the effects of anisotropy of the myocardium tissue in our results. This means that we have more than one independent shear and more than one independent Young’s modulus. Our material is not fully orthotropic with three Young’s moduli and three shears since there is a symmetry in *x* and *y*. So therefore due to the symmetries imposed by our choice of geometry we should note that the shear $C_{44}$ is the same as the shear $C_{55}$, so we consider shears $C_{44}$ and $C_{66}$. We also only have the two Young’s moduli $E_1$ and $E_3$, since $E_{1}$ is the same as $E_{2}$.

Here we compare the shear modulus $C_{44}$ for a healthy myocyte embedded in the extracellular matrix with a setup where there has been loss of myocyte volume fraction and increased fibrosis of the matrix designed to represent the case of myocardial infarction. The parameter $C_{44}$ is taken directly from the computed effective elasticity tensor for the model. We have plotted the comparison of the shear moduli for the healthy and infarcted cases over a range of porosities from 2–30%. This is shown in the figures below.

**Fig. 6:**
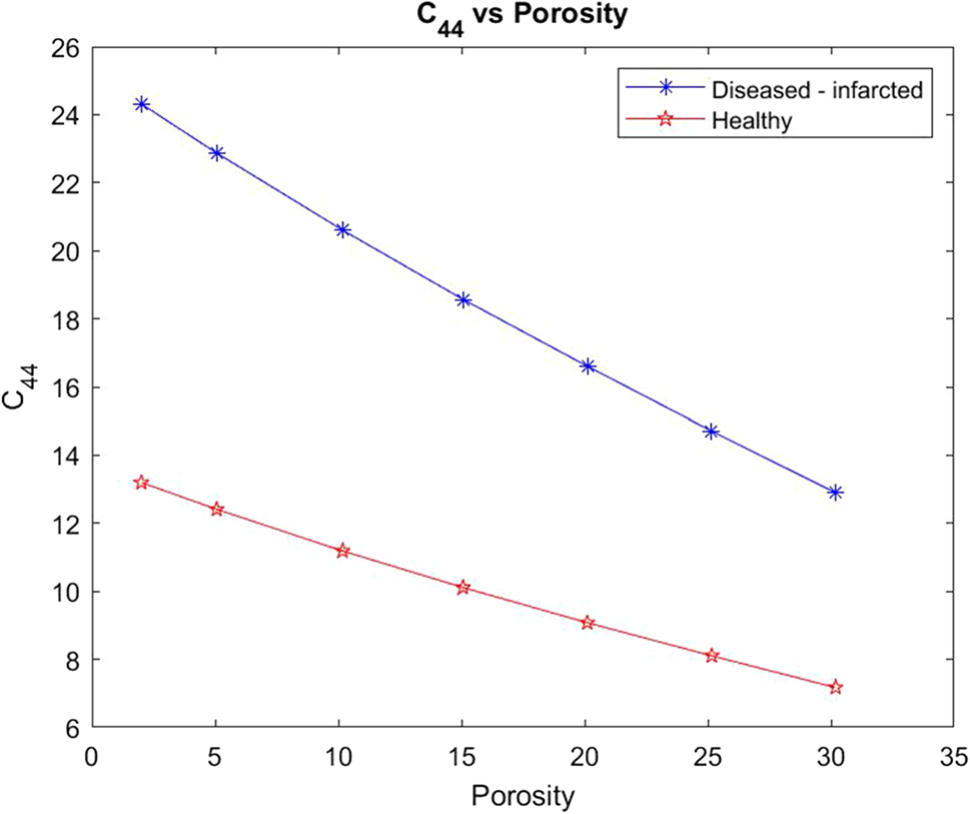
Shear $C_{44}$ versus porosity for both the healthy heart and the infarcted case

We see in Fig. 6 that the healthy setup has much lower values for shear and produces an overall smaller decrease in shear with increasing porosity than the diseased case. The shear is being applied in the axial direction (where the myocytes and voids elongate) so the material deforms into the voids and they flatten out allowing for the decrease in shear as the voids increase in size (larger porosity). The diseased case has a higher initial value for shear due to the increased stiffness of the matrix and the unusual geometry of the damaged myocyte, compared to the healthy case which has the normal soft extracellular matrix and regular myocyte. The higher the shear the stiffer the overall material, this means in the case of infarction even with reperfusion (increase in porosity) the stiffness of the myocardium still does not return to the normal healthy value. However, the increased perfusion does improve the overall compliance of the diseased material.

We also carry out the same comparison but this time for the shear modulus $C_{66}$.

**Fig. 7:**
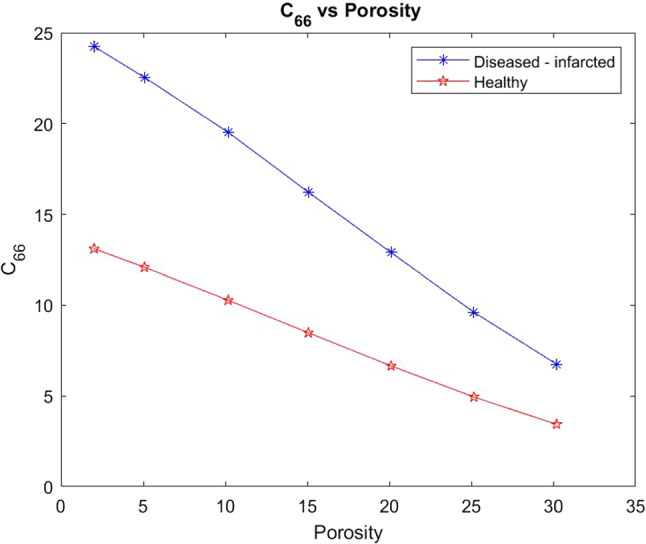
Shear $C_{66}$ versus porosity for both the healthy heart and the infarcted case

In Fig. 7 we see that the healthy setup begins with a much lower shear value even at small porosities compared with the infarcted case. The healthy case produces an overall much smaller decrease in shear with increasing porosity than the diseased case. The shear is being applied in the *x*-direction (transverse). Therefore for both the diseased and healthy cases the force is being applied taking a cross section of structure which contains the voids and the myocyte. At higher porosities this makes the material weaker as the larger voids deform more easily hence why the decrease in shear is observed in both cases. The diseased case has a higher initial value for shear $C_{66}$ due to the increased stiffness of the matrix and the unusual geometry of the damaged myocyte, compared to the healthy case which has the normal soft extracellular matrix and regular myocyte. Again the increase in porosity (to mimic reperfusion) in the diseased case does reduce the overall stiffness of the material to attempt to return to a similar stiffness as the healthy. Comparing the shear $C_{66}$ with shear $C_{44}$ we can see that $C_{66}$ has higher initial values but with increasing porosity actually becomes lower than $C_{44}$. This can be explained by the geometry and the direction in which the myocytes elongate and the presence of the voids. The voids have the larger influence on shear when applying in the $C_{66}$ direction as they deform easily with less influence from the myocyte.

We also wish to consider the comparison between the two Young’s moduli $E_{1}$ (transverse) and $E_{3}$ (axial) for the healthy and the infarcted heart using the LMRP model. We compute the components of the effective elasticity tensor for both the healthy and infarcted cases and use in the formulas for the Young’s moduli. These formulas, which can be derived via inverting the elasticity tensor and comparing with the material compliance tensor (Vignjevic et al. 2008), are given by

19 $$\begin{aligned} E_{1}&=\frac{(C_{12}-C_{11})(2C_{13}^{2}-C_{12}C_{33}-C_{11}C_{33})}{(-C^2_{13}+C_{11}C_{33})}\end{aligned}$$

20 $$\begin{aligned} E_{3}&=\frac{(2C^2_{13}-C_{12}C_{33}-C_{11}C_{33})}{(-C_{12}-C_{11})} \end{aligned}$$

We plot the comparison of Young’s moduli between the healthy and infarcted cases.

**Fig. 8:**
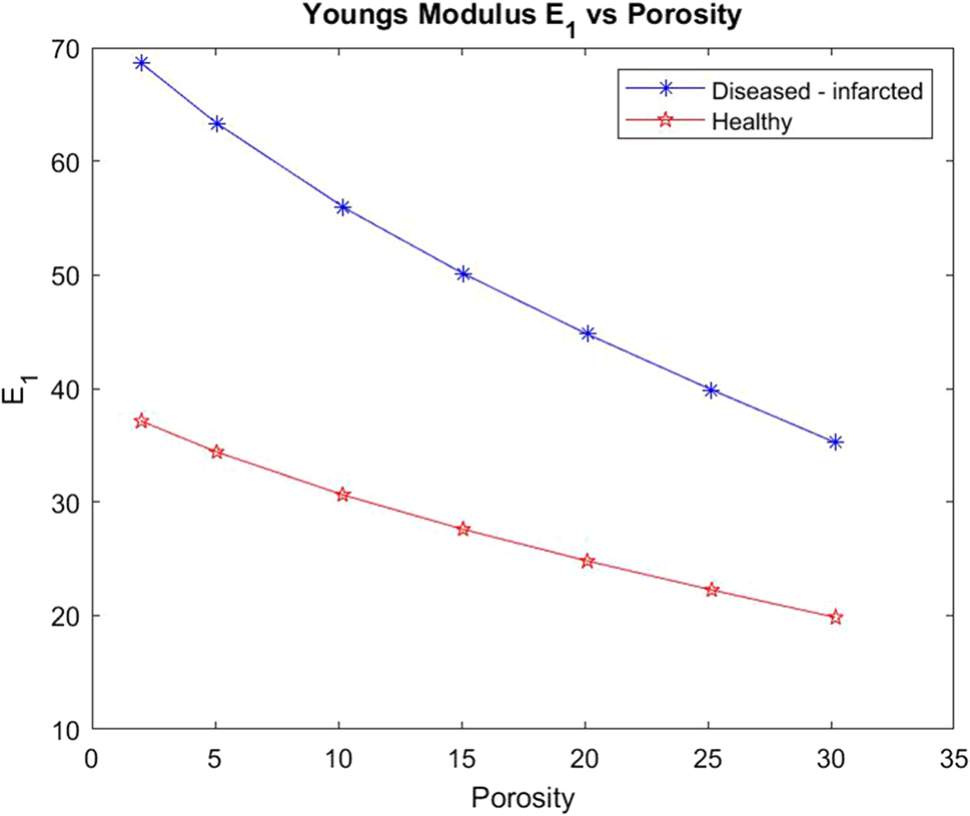
$E_{1}$ versus porosity for both the healthy heart and the infarcted case

Figure 8 shows that the infarcted myocardium has a much higher transverse Young’s modulus than the healthy case (almost double the stiffness). This is explained by the fact that the matrix in the infarcted case is much stiffer than in the healthy case and therefore influences the overall stiffness of the material to a large extent. The infarcted case also has the damaged myocyte which has lost volume and been replaced by the stiffer matrix which also influences the overall stiffness of the myocardium. We see that the stiffness of the infarcted case reduces dramatically with increasing porosity of the material. This means that with reperfusion of the infarcted tissue then the stiffness of the myocardium can be reduced with the benefit that the overall compliance of the tissue will then improve, thus improved heart function. We do see however that even at the highest porosities the diseased case never reaches the standard healthy $E_1$ value that would be approximately 30 kPA.

**Fig. 9:**
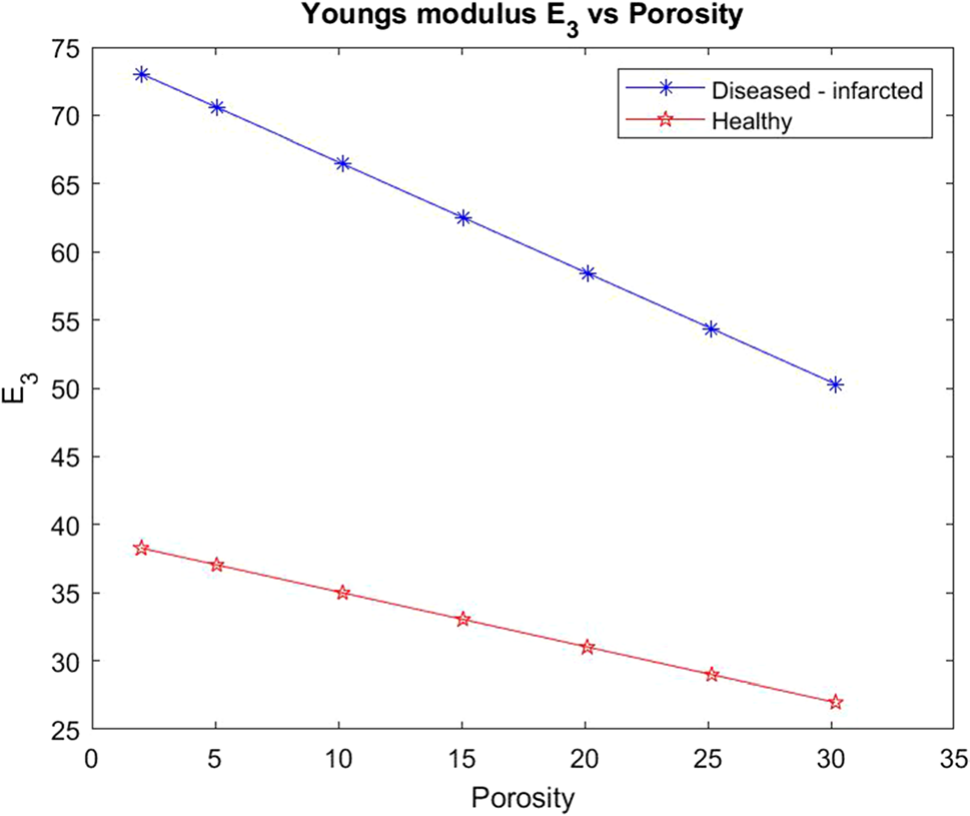
$E_{3}$ versus porosity for both the healthy heart and the infarcted case

In Fig. 9 we consider the Young’s modulus $E_3$. We can again see that the healthy myocardium has a much lower axial Young’s modulus than the infarcted case. In fact the infarcted Young’s modulus is approximately double that of the healthy case. Overall again the increasing porosity does have an effect in reducing the overall stiffness in both cases with the effects of the increasing porosity being shown more clearly in the diseased case. The increasing porosity has a much greater effect on the infarcted case in an attempt to improve the compliance of the overall heart muscle. We can compare $E_3$ with $E_1$. We find that both the healthy and infarcted axial Young’s moduli ($E_3$) are initially higher than both the healthy and infarcted transverse Young’s moduli ($E_1$). We can see that the infarcted $E_3$ is always higher than the infarcted $E_1$. We can also observe that the healthy $E_3$ is also always higher than the healthy $E_1$. This is due to the fact that the myocytes and voids elongate in the axial direction which is also considered a contributory factor to the stiffness in that direction.

## Changing myocyte volume fraction

Following myocardial infarction we see a decrease in the volume fraction of myocytes in the infarct zone due to the death and damage of myocytes, however, in the regions surrounding the infarct zone the intact myocytes increase in volume to attempt to compensate for the section of damaged heart (Olivetti et al. 1987). We therefore wish to investigate the influence that this change in volume has on the overall elastic parameters of the heart. We assume our increase in myocyte volume fraction corresponds to different infarct sizes and not a time dependent increase following the infarction (Olivetti et al. 1994; Anversa et al. 1985).

Within this section, we make the assumption that the myocytes run from the top of the cell to the bottom as a single cylindrical fibre. The myocytes here are intact cylinders since they have not been damaged by the infarction. This means that we can cut the plane and perform 2D simulations to solve the cell problems of LMRP. For a description of the cell problems see Appendix A and for the complete 2D reduction of the model see (Miller and Penta 2022).

We solve the cell problems using the following parameters, found in Andreu et al. (2014); Chiou et al. (2016); Lieber et al. (2004), summarised in the table below (Table 2).

**Table 2:** Input parameters

Parameter	$E_{\textrm{myo}}$	$E_{\textrm{matrix}}$	$\nu _{\textrm{myo}}$	$\nu _{\textrm{matrix}}$
Value	35	80	0.49	0.4

We carry out the simulations for four fixed fluid volume fractions $\phi _{\text {f}}=5\%, 10\%, 15\%, 20\%$ and for each of these varying the myocyte volume fraction from 5–30%. The fluid volume fractions have been chosen to represent the following settings; 5% reduced flow leading to infarction, 10–15% normal range of healthy perfusion, 20% over perfused leading to myocardial injury.

We begin by considering the two Young’s moduli $E_{1}$ and $E_{3}$ for the infarcted heart.

**Fig. 10:**
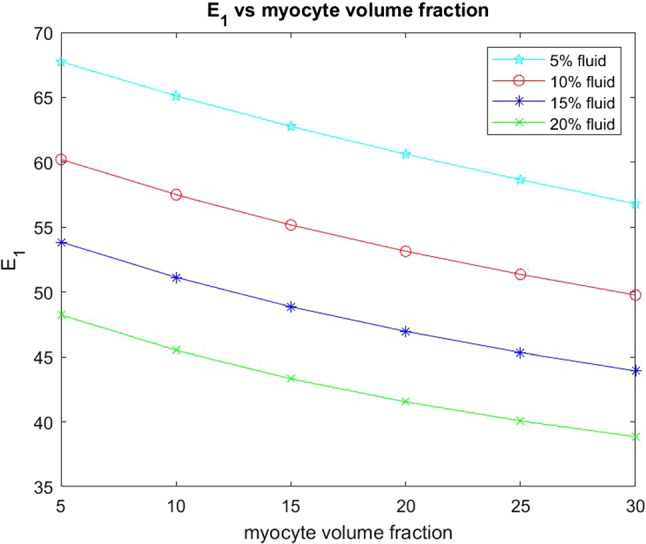
$E_1$ versus myocyte volume fraction for four different fixed fluid volume fractions

In Fig. 10 we see that the transverse Young’s modulus $E_1$ decreases with increasing myocyte volume fraction and this behaviour is consistent across the four fixed fluid volume fractions that we have considered. The Young’s modulus can be thought of as a measure of material stiffness so in the case of low myocyte volume fraction the extracellular matrix is the dominating parameter in influencing the stiffness of the overall material. A stiffer material leads to less elastic compliance which can be detrimental for overall function of the heart. This is why in the regions surrounding a myocardial infarction the myocyte volume fractions increase as their increase in volume actually reduces the overall stiffness and hence improves the overall compliance of the material. This biological mechanism is highlighted in the results of our simulations.

We also wish to consider the axial Young’s modulus $E_3$. This Young’s modulus is in the same direction that the myocytes and voids elongate.

**Fig. 11:**
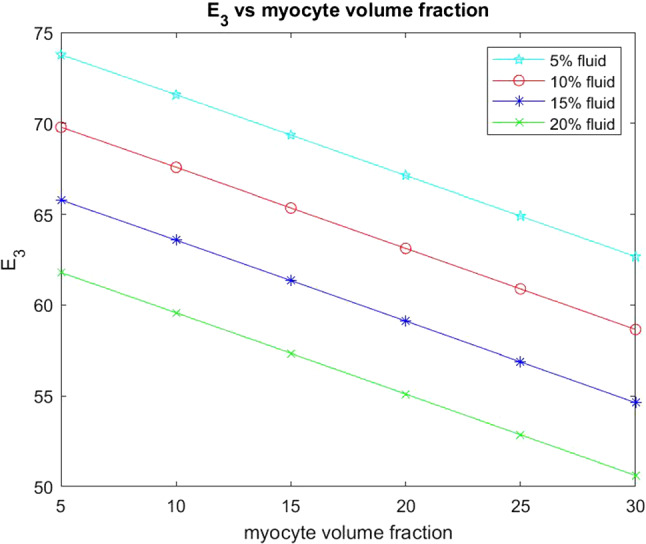
$E_3$ versus myocyte volume fraction for four different fixed fluid volume fractions

In Fig. 11 we see that the axial Young’s modulus $E_3$ also decreases with increasing myocyte volume fraction and this behaviour is consistent across the four fixed fluid volume fractions that we have considered. In the case of $E_3$ the values are higher for each of the fixed fluid volume fractions when compared to the transverse Young’s modulus $E_1$. This is due to the fact that the myocytes elongate in this direction which adds to the increased stiffness. Again since the matrix is stiffer as a result of the myocardial infarction then the increase in myocyte volume fraction helps to reduce the stiffness and improve the compliance of the material, which again emphasises the observed physiological response.

The other two elastic parameters that we consider for varying myocyte volume fraction are the shear moduli $C_{44}$ and $C_{66}$. These parameters are taken directly from the computed effective elasticity tensor for the model. In the same way as with the Young’s moduli we have plotted the comparison of the shear moduli over a range of myocyte volume fractions from 5–35% at the four fixed fluid volume fractions. This is shown in the figures below.

**Fig. 12:**
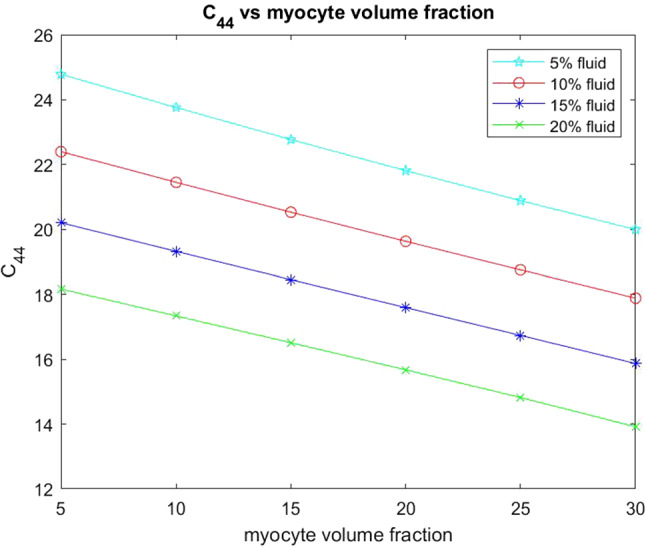
Shear $C_{44}$ versus myocyte volume fraction for four different fixed fluid volume fractions

From Fig. 12 we can see that the shear $C_{44}$ decreases with increasing myocyte volume fraction. In the case of $C_{44}$ the force is being applied in the axial direction, this is the direction in which the myocytes and blood vessels elongate. The blood vessels can be thought of as empty channels since we are considering the drained parameters. This means that when the force is applied to the material it deforms and the channels flatten out. This means that the empty channels just make it softer allowing for the decrease in shear with the increasing fluid volume fraction. When the myocytes have a low volume fraction, such as in the case where myocyte damage and death has occurred due to myocardial infarction, then we see that, for all four fixed fluid volumes, the shear values are higher than for a larger myocyte volume fraction. The stiffest scenario is for fixed $5\%$ fluid volume and low myocyte volume fraction and this can be representative of the situation directly following myocardial infarction where fluid flow to the tissue has been dramatically reduced resulting in the loss of myocyte volume. We see that by increasing the myocyte volume fraction the shear decreases at all four fluid volumes, meaning that we have a softer and more compliant material once the myocytes increase in size. Physiologically this occurs to help the myocardium return to homeostasis after infarction and this mechanism is clearly observed from our simulations.

**Fig. 13:**
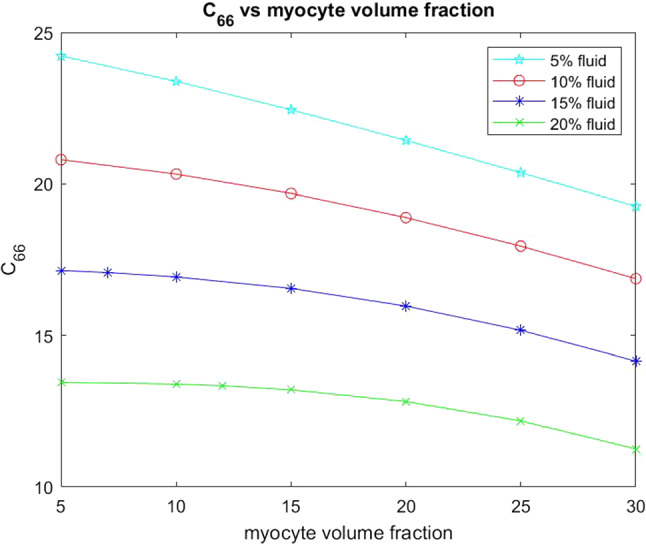
Shear $C_{66}$ versus myocyte volume fraction for four different fixed fluid volume fractions

In Fig. 13 the shear $C_{66}$ decreases with increasing myocyte volume fraction for all four fixed fluid volumes. For $C_{66}$ the force is being applied in the transverse direction, that is, the force is being applied taking a cross section of the structure where we have the myocyte and the channels. At the lowest fluid volume fraction and smallest myocyte volume (the scenario representing immediately post myocardial infarction) we see that the shear is the largest, this means that under this setting the myocardium is very stiff. The typical healthy shear for the myocardium would be approx. 10 kPa which is much lower that the 24.2 kPa value we see for the infarcted setup. This motivates the myocardium’s biological response to increase the myocyte volume fraction in order to try to return the tissue to the correct shear values so that the stiffness and compliance of the material are closer to the healthy case which leads to greater efficiency of the recovered muscle. If we compare $C_{66}$ with $C_{44}$ we see that $C_{44}$ has the higher values across the increasing myocyte volume fraction. This is due to the fact that the shear $C_{44}$ is being applied in the direction the myocyte elongates so the increase in its volume influences the material in that direction making it stiffer.

## 3D simulations results—intercalated disks

**Fig. 14:**
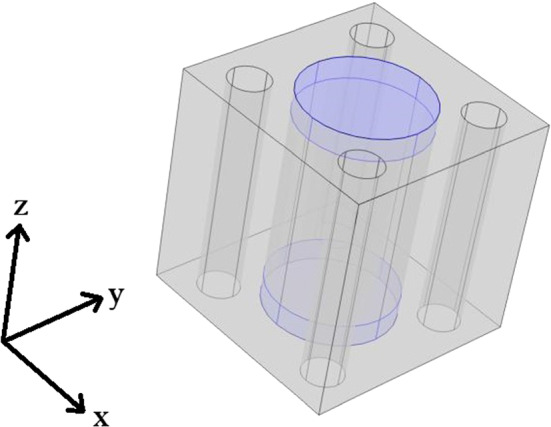
3D geometry myocyte with intercalated disks at both ends embedded in the extracellular matrix with four blood vessels

Within this section we extend the current computational platform to 3D to allow us incorporate more structural details that will give us an even more detailed picture of the true elastic response of the heart. We now consider a setup where we have the myocyte with intercalated disks at either end embedded in the extracellular matrix with the four blood vessels in each corner. The intercalated disks are thin connecting plates found at either end of the myocytes that allow for connection to the next myocyte cell (Moise et al. 2021). The more detailed 3D geometry we consider is shown in Fig. 14.

Here we make the assumption that the myocytes have a height of 0.8 in the unit cell of length 1. This is centred so there is a gap of 0.1 height between the myocyte ends and the top and bottom of the cell. In this gap we place the intercalated disks that connect the myocytes between cells. This means that we must perform 3D simulations to solve the cell problems of LMRP since for every *z* we do not have the same microstructure so we cannot reduce to 2D.

We solve the cell problems using the following parameters, found in Andreu et al. (2014); Chiou et al. (2016) and Lieber et al. (2004). As far as can be determined from the literature there is no clear Young’s modulus for the intercalated disk, this can be attributed to the fact they are composed of a variety of different proteins all with different elastic parameters. However, we do know that the intercalated disks between myocytes are exposed to substantially higher forces than the equivalent cell-cell junctions in other organs (McCain et al. 2012). This leads to the assumption that disks should be stiffer than the myocyte but on the same order of magnitude. The myocardium also becomes stiffer in the case of hypertrophic cardiomyopathy due to an accumulation of proteins in the intercalated disks (Masuelli et al. 2003). We therefore have the following parameters and the values we have selected for the intercalated disks shown in (Table 3).

**Table 3:** Input parameters 3D simulations

Parameter	$E_{\textrm{myo}}$	$E_{\textrm{matrix}}$	$E_{\textrm{disk}}$	$\nu _{\textrm{myo}}$	$\nu _{\textrm{matrix}}$	$\nu _{\textrm{disk}}$
Value	35	80	60	0.49	0.4	0.49

We carry out the simulations for four fixed fluid volume fractions $\phi _{\text {f}}=5\%, 10\%, 15\%, 20\%$ and for each of these varying the myocyte volume fraction from 5–25%. The fluid volume fractions have been chosen to represent the following settings; 5% reduced flow leading to infarction, 10–15% normal range of healthy perfusion, 20% over perfused leading to myocardial injury. We should note that the intercalated disk is the connection between the myocytes, therefore we are assuming that the intercalated disks are growing with the myocytes so that the radii of both are consistently the same. This means that we are losing a larger percentage of the matrix with the increase in myocyte volume fraction at the expense of the larger disks.

We begin our analysis by considering the Young’s moduli.

**Fig. 15:**
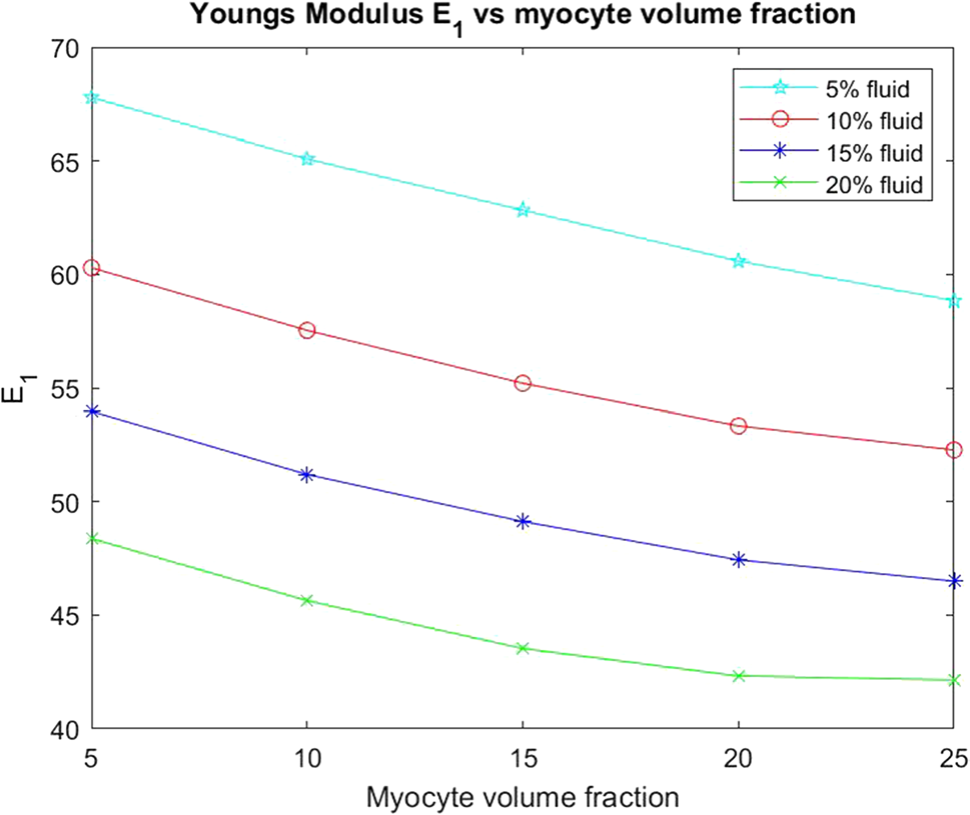
Young’s Modulus $E_{1}$ versus myocyte volume fraction for four different fixed fluid volume fractions

From Fig. 15 we can see that for each fixed fluid volume fraction that the Young’s modulus $E_1$ decreases with increasing myocyte volume fraction. As before the Young’s modulus is a measure of the material stiffness and therefore gives information about the overall elastic compliance of the heart. The heart should be soft and elastic when healthy with an overall Young’s modulus of 35 kPA (Lieber et al. 2004). This means we can determine a range of conclusions from the simulations that agree with physiological findings. Post myocardial infarction intact, surviving myocytes enlarge in an attempt to regulate the stiffness of the heart caused by the increasing stiffness of the extracellular matrix. Here we see exactly this phenomena, the larger the myocyte volume and the greater the fluid volume fraction the closer the $E_1$ parameter gets to that of the healthy heart.

**Fig. 16:**
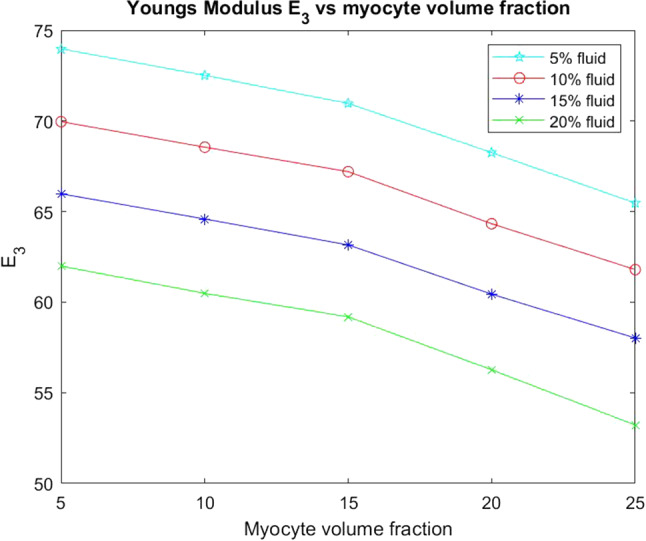
Young’s Modulus $E_{3}$ versus myocyte volume fraction for four different fixed fluid volume fractions

Here, in Fig. 16, we consider the axial Young’s modulus $E_3$. We again can see that with increasing myocyte volume fraction the value of $E_3$ (the stiffness) decreases for all four fixed fluid volumes. We again see that this behaviour is again representative of what happens physiologically in the heart to try to maintain homeostasis post infarction. We also can compare $E_3$ with $E_1$. We see that $E_3$ changes more than $E_1$ when we compare line-by-line (for each fluid volume fraction) and that the starting values of $E_3$ are higher than that of $E_1$. This can be explained by the fact that since the myocytes elongate in $E_3$ this creates the stiffer Young’s modulus in this direction compared with the $E_1$ Young’s modulus.

We also wish to consider the two shear moduli $C_{44}$ and $C_{66}$ for the four fixed fluid volumes with increasing myocyte volume.

**Fig. 17:**
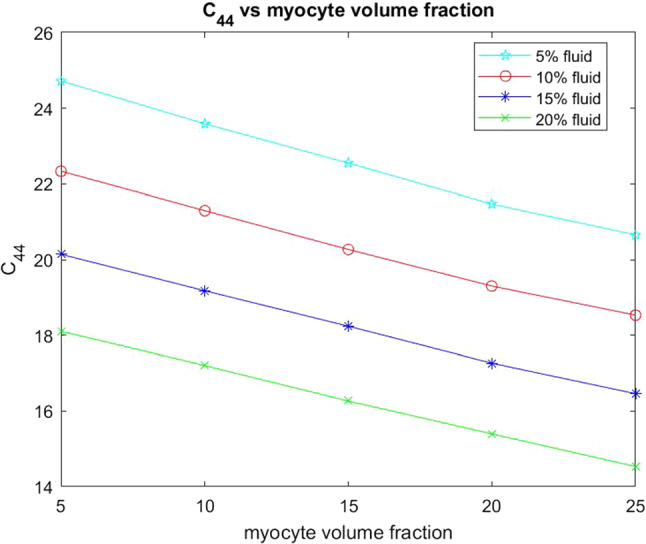
Shear $C_{44}$ versus myocyte volume fraction for four different fixed fluid volume fractions

In Fig. 17 we see that the shear $C_{44}$ decreases with increasing myocyte volume fraction at all four fluid volumes. This shear is applied in the direction that the myocyte and channels elongate. This means that for small myocyte volume fractions the matrix and the specified fluid volume fraction have most influence on the stiffness of the material. When the myocytes and disks increase in volume they play a role in reducing the overall stiffness since they are softer than the matrix. The higher the value of the shear then the stiffer the overall material is. The results of our simulations again agree with the physiologically observed behaviour that the increased myocyte volume aims to reduce the overall stiffness of the myocardium caused by the infarct scar in an attempt to return to homeostasis.

**Fig. 18:**
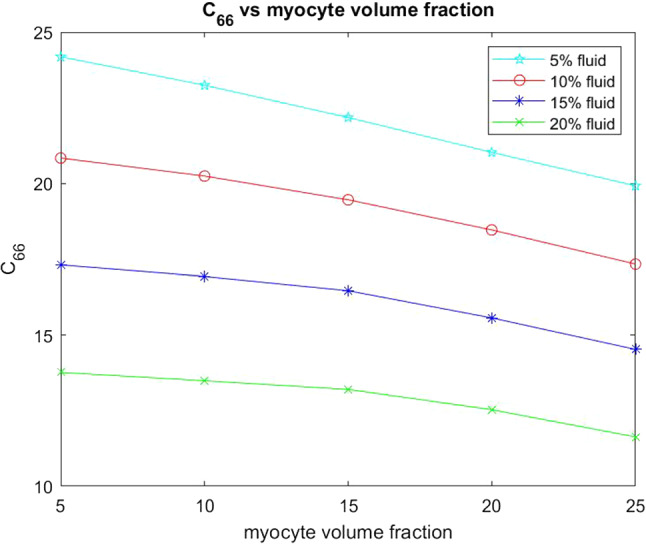
Shear $C_{66}$ versus myocyte volume fraction for 4 different fixed fluid volume fractions

The final parameter we have considered is the shear $C_{66}$ as shown in Fig. 18. This shear is applied taking a cross-section of the material where we will see matrix, channels and intercalated disk. This shear again decreases with increasing myocyte volume. We can compare the behaviour with $C_{44}$. We see that for the 5% fluid volume fraction that $C_{66}$ decreases more than $C_{44}$, however for 10%, 15% and 20% fluid volume fractions $C_{44}$ shows the greater decrease with increasing myocyte volume fraction. We note that $C_{44}$ has higher values across all myocyte volume fractions for all four fluid volume fractions than $C_{66}$. This can be explained by the different directions the shear is applied in. The increase in the myocyte volume does indeed decrease the overall stiffness since the myocyte and the disks are taking up a larger volume of the whole structure and are softer than the matrix. However $C_{44}$ is being applied against the base of the disk/myocyte fibre and as the volume of this increases it has an influence when the force is applied. The voids with the $C_{44}$ shear only flatten out rather than deforming with the shear. Both of these reasons are what keeps the value of the shear $C_{44}$ higher that that of $C_{66}$.

## Conclusions and future directions

Within this work we have created a robust computational platform that has allowed for a first study of how different microstructural features, that can be observed clinically following myocardial infarction, affect the elastic parameters of the heart. We have investigated a variety of elastic parameters obtained by solving the asymptotic homogenization cell problems of Miller and Penta (2020) for poroelastic composites.

We begin this work by firstly summarising the LMRP model for poroelastic composites. We provide an Appendix A with the specific cell problems that we have solved to produce the results of this work, as well as references to inform the reader of the numerical procedures that are carried out. We then consider the first microstructural change that occurs as the result of myocardial infarction. That is, the loss of myocyte volume and increasing matrix fibrosis and we consider this versus porosity (Prabhu and Frangogiannis 2016). For this microstructural change we make the comparison with the healthy heart for the four elastic parameters (Young’s moduli $E_1$ and $E_3$ and shear moduli $C_{44}$ and $C_{66}$). We find that in all cases the diseased/infarcted heart is much stiffer across the range of porosities considered. This is in line with the expected physiological response post infarction (Gupta et al. 1994; Voorhees and Han 2014).

We continue our analysis by considering the effect of increasing the volume fraction of the myocyte with the extracellular matrix still being stiffer than in the healthy case. Physiologically this happens in the areas surrounding the infarct region in an attempt to counter balance the increased stiffness of the matrix with scar tissue (Olivetti et al. 1987). The results we obtain for all four elastic parameters, for all four different fixed fluid volume fractions, confirms this physiological phenomenon (Olivetti et al. 1994). For both of these cases it was possible to carry out the simulations in 2D since our geometry is identical for each *z*.

The final part of our analysis extends the previous section by the addition of the intercalated disks that are stiffer than the myocytes and connect myocytes cell-to-cell. The analysis carried out in this section requires 3D simulations since the microstructure varies with the *z* coordinate. In this setting it is again the increase in the myocyte volume fraction that is considered. We again see that with increasing myocyte volume all of the elastic parameters that we have considered here decrease, meaning the stiffness of the overall myocardium is decreasing. Once again our numerical results were replicating the physiological response (i.e. increasing myocyte volume in order to try to reduce the stiffness of the complete organ caused by the scar tissue (Olivetti et al. 1987, 1994)).

The numerical simulations carried out in this work can be thought of as a first attempt to model some basic microstructural changes that can be observed post myocardial infarction. The simulations here are computationally cheap (approximately 15 s computing time to obtain each data point in the 3D and 2–3 s in the 2D) and can provide realistically observed physiological responses.

Our current model does have some limitations and possible extensions. The model currently assumes a simplified microstructure, we therefore could extend the 3D simulations in Sect. 5 for more complicated geometries. It would also be possible to split the heart into regions such as infarct zone, the infarct border and the remaining unaffected tissue. By doing this we would obtain different macroscale coefficients for each of the regions that can be used to solve the overall macroscale model. The solution of the macroscale model would be straight-forward to obtain and can be found by using a scheme similar to the one proposed in Penta et al. (2020). The macroscale model of standard Biot’s poroelasticity has been solved in Dehghani et al. (2020).

Currently this work has used linear elasticity however, we are able to make use of this computational platform to represent a more accurate nonlinear behaviour of the heart by using a piecewise approach to modelling as done in Hu et al. (2003a, 2003b). By doing this we can approximate the nonlinear behaviour using simple, computationally cheap simulations.

Future extensions to this model that could allow it to be used as a predictive tool for clinicians would be adding additional microstructural features that have an influence on the overall behaviour of the heart. The microstructures chosen in this work are a very basic starting point as a first approximation to the myocardial microstructure using the computational platform that we have created for our novel poroelastic composite model. The next steps will be to carry out further numerical tests with increasingly complex microstructures such as to include fibroblasts (Humeres and Frangogiannis 2019; Fan et al. 2012), different directions of collagen and elastin fibres (Purslow 2008; Ohayon and Chadwick 1988) and indeed tortuosity of the channels Penta and Ambrosi (2015). As well as obtaining additional data from medical imaging that would allow us to create a patient specific profile of the elastic parameters post infarction and in the recovery period.

Another useful source of data for model comparison and validation would come from elastography of the heart. The elastography technique uses vibrations applied to the skin and measures the responses from the underlying tissues since stiffer tissue responds differently to softer tissue (Wells and Liang 2011) This can be used to assess changes in myocardial elasticity during the cardiac cycle (Tzschätzsch et al. 2012). These measurements along with our model predictions can provide valuable insight to clinicians on the stiffness of the heart and inform treatment choices.

A final consideration that may be useful to our model simulations is the effects of the cell membranes of the different components in our periodic cell. This consideration can be very useful for some tissue types and perhaps less influential in others. Factors that could influence the importance of the cell membrane on the mechanics are the structure of the membrane and its stiffness or the permeability of the membrane. The cell membrane can have the following important mechanical properties such as its elastic modulus, shear modulus, bending stiffness, and viscosity (Dai and Sheetz 1997). Cell membranes have a very low shear modulus as well as a high elastic modulus. The permeability of the membrane influences the transport of substances across the cell membrane and in this case will be extremely important for the mechanics. This was investigated in Penta and Merodio (2017) for vascularised poroelastic materials.

## Appendix A Cell problems

Within this appendix we present the cell problems for poroelastic composites arising from the asymptotic homogenization technique as found in Miller and Penta (2020). These cell problems allow us to compute all of the macroscale model coefficients.

We are able to compute the fourth rank effective elasticity tensor $\widetilde{{\mathbb {C}}}$ for the LMRP model and by using its components calculate the two Young’s moduli and two shear moduli. The effective elasticity tensor is given by

A1 $$\begin{aligned} \widetilde{{\mathbb {C}}}=\langle \mathbb {C_{\scriptscriptstyle \textrm{Myo}}M_{\scriptscriptstyle \textrm{Myo}}} +\mathbb {C_{\scriptscriptstyle \textrm{Myo}}}+ \mathbb {C_{\scriptscriptstyle \textrm{M}}M_{\scriptscriptstyle \textrm{M}}} +\mathbb {C_{\scriptscriptstyle \textrm{M}}}\rangle _s. \end{aligned}$$

We can see that this comprises the fourth rank tensor ${\mathbb {M}}_i$, where $i=\textrm{Myo, M}$, which can be defined as

A2 $$\begin{aligned} {\mathbb {M}}_{\textrm{Myo}}= & {} \xi _{pq}^{kl}(A^{\scriptscriptstyle \textrm{Myo}})=\frac{1}{2}\bigg (\frac{\partial A^{\scriptscriptstyle \textrm{Myo}}_{pkl}}{\partial y_{q}}+\frac{\partial A^{\scriptscriptstyle \textrm{Myo}}_{qkl}}{\partial y_{p}}\bigg ); \quad \nonumber \\ {\mathbb {M}}_{\textrm{M}}= & {} \xi _{pq}^{kl}(A^{\scriptscriptstyle \textrm{M}})=\frac{1}{2}\bigg (\frac{\partial A^{\scriptscriptstyle \textrm{M}}_{pkl}}{\partial y_{q}}+\frac{\partial A^{\scriptscriptstyle \textrm{M}}_{qkl}}{\partial y_{p}}\bigg ). \end{aligned}$$

We can then write the cell problems for third rank tensors $A_{\scriptscriptstyle \textrm{Myo}}$ and $A_{\scriptscriptstyle \textrm{M}}$, found in Miller and Penta (2020), with corresponding components $A_{ikl}^{\scriptscriptstyle \textrm{Myo}}$ and $A_{ikl}^{\scriptscriptstyle \textrm{M}}$ as

A3 $$\begin{aligned}&\frac{\partial }{\partial y_{j}}\bigg (C^{\scriptscriptstyle \textrm{Myo}}_{ijpq}\xi _{pq}^{kl}(A^{\scriptscriptstyle \textrm{Myo}})\bigg )+\frac{\partial C^{\scriptscriptstyle \textrm{Myo}}_{ijkl}}{\partial y_j}=0 \qquad \quad \text{ in } \quad \Omega _{\scriptscriptstyle \textrm{I}}\end{aligned}$$

A4 $$\begin{aligned}&\frac{\partial }{\partial y_{j}}\bigg (C^{\scriptscriptstyle \textrm{M}}_{ijpq}\xi _{pq}^{kl}(A^{\scriptscriptstyle \textrm{M}})\bigg )+\frac{\partial C^{\scriptscriptstyle \textrm{M}}_{ijkl}}{\partial y_j}=0 \qquad \quad \text{ in } \quad \Omega _{\scriptscriptstyle \textrm{II}}\end{aligned}$$

A5 $$\begin{aligned}&C^{\scriptscriptstyle \textrm{Myo}}_{ijpq}\xi _{pq}^{kl}(A^{\scriptscriptstyle \textrm{Myo}}) n^{\scriptscriptstyle \textrm{III}}_j-C^{\scriptscriptstyle \textrm{M}}_{ijpq}\xi _{pq}^{kl} (A^{\scriptscriptstyle \textrm{M}})n^{\scriptscriptstyle \textrm{III}}_j=(C^{\scriptscriptstyle \textrm{M}} -C^{\scriptscriptstyle \textrm{Myo}})_{ijkl}n^{\scriptscriptstyle \textrm{III}}_j \qquad \quad \text{ on } \quad \Gamma _{\scriptscriptstyle \textrm{III}}\end{aligned}$$

A6 $$\begin{aligned}&A^{\scriptscriptstyle \textrm{Myo}}_{ikl}=A^{\scriptscriptstyle \textrm{M}}_{ikl} \qquad \quad \text{ on } \quad \Gamma _{\scriptscriptstyle \textrm{III}}\end{aligned}$$

A7 $$\begin{aligned}&C^{\scriptscriptstyle \textrm{M}}_{ijpq}\xi _{pq}^{kl}(A^{\scriptscriptstyle \textrm{M}})n^{\scriptscriptstyle \textrm{II}}_j +C^{\scriptscriptstyle \textrm{M}}_{ijkl}n^{\scriptscriptstyle \textrm{II}}_j=0\qquad \quad \text{ on } \quad \Gamma _{\scriptscriptstyle \textrm{II}} \end{aligned}$$

The solutions to the problem (A3–A7), $\xi _{pq}^{kl}(A^{\scriptscriptstyle \textrm{Myo}})$ and $\xi _{pq}^{kl}(A^{\scriptscriptstyle \textrm{M}})$, are found by solving six elastic-type cell problems by fixing the couple of indices (*k*, *l*). By doing this the $\xi _{pq}^{kl}(A^{\scriptscriptstyle \textrm{Myo}})$ and $\xi _{pq}^{kl}(A^{\scriptscriptstyle \textrm{M}})$ that appear in (A3–A7) represent a strain, Then for every fixed couple (k, l) we have a linear elastic problem which has interface conditions between the matrix and inclusion determined by using the elasticity tensor in (A5) and between the matrix and the fluid that can be determined using (A7).

We also wish to be able to determine the other macroscale coefficients such as the Biot’s modulus and the Biot’s tensor of coefficients (see 17). We see that these coefficients contain the tensors ${\mathbb {Q}}_{\textrm{Myo}}$ and ${\mathbb {Q}}_{\textrm{M}}$. These can be defined as

A8 $$\begin{aligned} {\mathbb {Q}}_{\textrm{Myo}}= & {} \xi _{pq}(\mathbf{{a}}^{\scriptscriptstyle \textrm{Myo}})=\frac{1}{2}\bigg (\frac{\partial {{a}}^{\scriptscriptstyle \textrm{Myo}}_{p}}{\partial y_{q}}+\frac{\partial {{a}}^{\scriptscriptstyle \textrm{Myo}}_{q}}{\partial y_{p}}\bigg ); \quad \nonumber \\ {\mathbb {Q}}_{\textrm{M}}= & {} \xi _{pq}(\mathbf{{a}}^{\scriptscriptstyle \textrm{M}})=\frac{1}{2}\bigg (\frac{\partial {{a}}^{\scriptscriptstyle \textrm{M}}_{p}}{\partial y_{q}}+\frac{\partial {{a}}^{\scriptscriptstyle \textrm{M}}_{q}}{\partial y_{p}}\bigg ). \end{aligned}$$

We then have the cell problems for the vectors $\mathbf{{a}}^{\scriptscriptstyle \textrm{Myo}}$ and $\mathbf{{a}}^{\scriptscriptstyle \textrm{M}}$, which is given by

A9 $$\begin{aligned} \frac{\partial }{\partial y_j}\bigg (C^{\scriptscriptstyle \textrm{Myo}}_{ijpq}\xi _{pq}(\textbf{a}^{\scriptscriptstyle \textrm{Myo}})\bigg )=0&\qquad \quad \text{ in } \quad \Omega _{\scriptscriptstyle \textrm{I}}\end{aligned}$$

A10 $$\begin{aligned} \frac{\partial }{\partial y_j}\bigg (C^{\scriptscriptstyle \textrm{M}}_{ijpq}\xi _{pq}(\textbf{a}^{\scriptscriptstyle \textrm{M}})\bigg )=0&\qquad \quad \text{ in } \quad \Omega _{\scriptscriptstyle \textrm{II}}\end{aligned}$$

A11 $$\begin{aligned} C^{\scriptscriptstyle \textrm{Myo}}_{ijpq}\xi _{pq}(\textbf{a}^{\scriptscriptstyle \textrm{Myo}})n^{\scriptscriptstyle \textrm{III}}_j=C^{\scriptscriptstyle \textrm{M}}_{ijpq}\xi _{pq}(\textbf{a}^{\scriptscriptstyle \textrm{M}})n^{\scriptscriptstyle \textrm{III}}_j&\qquad \quad \text{ on } \quad \Gamma _{\scriptscriptstyle \textrm{III}}\end{aligned}$$

A12 $$\begin{aligned} a_i^{\scriptscriptstyle \textrm{Myo}}=a_i^{\scriptscriptstyle \textrm{M}}&\qquad \quad \text{ on } \quad \Gamma _{\scriptscriptstyle \textrm{III}}\end{aligned}$$

A13 $$\begin{aligned} C^{\scriptscriptstyle \textrm{M}}_{ijpq}\xi _{pq}(\textbf{a}^{\scriptscriptstyle \textrm{M}})n^{\scriptscriptstyle \textrm{II}}_j +n^{\scriptscriptstyle \textrm{II}}_j=0&\qquad \quad \text{ on } \quad \Gamma _{\scriptscriptstyle \textrm{II}} \end{aligned}$$

The solutions to the problem (A9–A13), $\xi _{pq}(\mathbf{{a}}^{\scriptscriptstyle \textrm{Myo}})$ and $\xi _{pq}(\mathbf{{a}}^{\scriptscriptstyle \textrm{M}})$, are found by solving the linear elastic problem with inhomogeneous Neumann conditions between the matrix and the fluid and continuity of auxiliary stresses between the two elastic phases.

These are the 3D cell problems and the ones used to compute the elastic parameters in Sect. 5. We must use the 3D problems when we have a variation in the *z* direction (i.e. in Sect. 5 we have cross-sections in the *z* direction that are disk and matrix or myocyte and matrix since this is different we must use the 3D problems). If the *z* cross-sections are all the same then it is possible to reduce these cell problems to 2D, which reduces the computational complexity. It is the reduced cell problems that are used in Sects. 3 and 4. For the complete detailed reduction of these cell problems to 2D please see (Miller and Penta 2022).
